# The Plant Direct Associate Reviewer Board

**DOI:** 10.1002/pld3.76

**Published:** 2018-07-18

**Authors:** Ivan R. Baxter

At *Plant Direct,* we take our role as a society journal seriously. So seriously, in fact, that we belong to *two* societies: the American Society of Plant Biologists and the Society for Experimental Biology. Part of our commitment is to publish the work of our community members without worrying about impact or novelty, as long as it meets our sound science criteria.

But that is not the only way we want to contribute to our community. We also want to be involved in training the next generation of plant scientists. The essential function of a journal—peer review—is conducted by our community members, but it is a skill that needs to be developed. Participating in peer review is a great opportunity for early career researchers to play a role in the community. It is a highly rewarding experience that can also improve a reviewer's own research and writing, as well as help to build their professional reputation. *Plant Direct* is a great venue for reviewer training, as the sound science criteria are easier for novice (and experienced) reviewers to judge than the more subjective novelty/impact criteria used at other journals.

So today, we are announcing the *Plant Direct* Associate Reviewer Board. We are looking to identify two groups of early career researchers: Those who are just beginning to review (upper‐level graduate students and beginning postdocs) and those who have performed reviews already and are looking to get more experience. The former group will receive training that is a combination of online training and hands‐on mentoring from *Plant Direct*'s Editorial Board. Once completed, they will be employed as additional reviewers for *Plant Direct* papers, while getting feedback on their reviews from the Supervising and Academic editors handling the paper. The latter group will be added to our reviewer pool with a special tag that lets our editors know that they are excited to review for us, a characteristic that all editors appreciate.

So if you are an early career researcher interested in developing and using your peer review skills, please join the Associate Reviewer Board [https://onlinelibrary.wiley.com/page/journal/24754455/homepage/associate-reviewer-board].

